# Exploring Farmers’ Climate Change Perceptions and Adaptation Intentions: Empirical Evidence from Austria

**DOI:** 10.1007/s00267-019-01158-7

**Published:** 2019-04-16

**Authors:** Hermine Mitter, Manuela Larcher, Martin Schönhart, Magdalena Stöttinger, Erwin Schmid

**Affiliations:** Institute for Sustainable Economic Development, Department of Economics and Social Sciences at the University of Natural Resources and Life Sciences, Vienna, Feistmantelstrasse 4, 1180 Vienna, Austria

**Keywords:** Farmers’ perceptions and adaptation intentions, Socio-cognitive process, Agricultural climate change adaptation, Adaptation avoidance, Socio-environmental context factors, Qualitative analysis

## Abstract

The lack of timely adaptation in agriculture may hamper prosperous farm developments by neglecting risks and opportunities emerging from climate change. Understanding farmers’ perceptual and socio-cognitive processes is key in order to encourage on-farm adaptation. We aim at investigating how farmers’ individual cognition on climate change and adaptation as well as socio-environmental context factors affect agricultural adaptation intention and avoidance. We build on the Model of Private Proactive Adaptation to Climate Change (MPPACC) and apply a qualitative interview approach in two Austrian farming regions. Twenty semi-structured interviews have been conducted with 29 farmers. Based on the results of the qualitative content analysis, we have identified four groups of farmers, which differ in the formation process of adaptation intention and avoidance: (i) climate change adaptors, (ii) integrative adaptors, (iii) cost-benefit calculators, and (iv) climate change fatalists. Farmers who are part of groups (i)–(iii) form adaptation intentions, whereas climate change fatalists do not intend to adapt. According to our analysis, adaptation intentions are only formed if farmers are aware of effective adaptation measures, accept personal responsibility for their farms, and evaluate adaptation costs positively (i.e. adaptation appraisal). Farmers’ climate change appraisal as well as farm and regional characteristics are also perceived relevant for farmers’ adaptation decisions but seem to be less important than adaptation appraisal. Therefore, we conclude that engagement strategies and outreach efforts need not only address risks and opportunities, but should also strengthen farmers’ self-responsibility and offer adaptation measures tailored to the regional characteristics and the farmers’ needs.

## Introduction

Agriculture is one of the most climate-sensitive sectors and directly affected by changes in physical and chemical climate conditions. Farmers may implement climate change adaptation measures to reduce or avoid adverse developments and take advantage of emerging opportunities. Others may forbear to adapt which results in a lack of timely adaptation. Farmers’ adaptation decisions—such as other human behaviour—underlie perceptual and socio-cognitive processes that are interwoven and refer to selecting, organizing, and interpreting sensory information. Socio-cognitive processes consider that human cognition also depends on the broader socio-environmental context, which may differ by culture and geographic region and may change over time (Grothmann and Patt 2005; Kastanakis and Voyer 2014; Risbey et al. 1999).

The relevance of perceptual and socio-cognitive processes for climate change adaptation in agriculture are underlined by the fact that farmers’ adaptation decisions take place at local level and respond to perceived socio-environmental contexts including, for instance, social and institutional support, cultural values and norms, regional characteristics, and climate-related trigger events (Grothmann and Patt 2005; Mitter et al. 2018). Against this background, perceptual and socio-cognitive processes have been investigated for farmers working in different cultural contexts and geographic regions, including major farming regions in North America (e.g. Arbuckle Jr et al. 2013; Doll et al. 2017; Haden et al. 2012; Mase et al. 2017; Nicholas and Durham 2012), South America (e.g. Frank et al. 2011), Europe (Alcon et al. 2014; Otto-Banaszak et al. 2011; Woods et al. 2017), Asia (Abid et al. 2015; Burnham and Ma 2017; Dang et al. 2014; Soriano et al. 2017), Africa (Mulenga et al. 2017), and Australia and New Zealand (Niles et al. 2016; Sanderson and Curtis 2016). The empirical results support the necessity to investigate and evaluate these processes within particular cultural and geographic contexts. For instance, Sanderson and Curtis 2016 show that cultural factors influence farmers’ climate change risk perception, which inform their adaptation decisions. Such detailed analyses for specific cultures and regions also contribute to expand and extend the knowledge on climate change adaptation in agriculture and establish the basis for subsequent integrative research.

In our research, we focus on Austrian farmers because of three reasons. First, climate change impacts may threaten Austrian farmers differently than their peers in other agro-environmental zones because climate change projections for the alpine region are particularly uncertain. Such uncertainties can decrease farmers’ intentions to adapt (Spence et al. 2012) and are mostly due to Austria’s location in a transition zone between rising winter precipitation in northern and declining summer precipitation in southern Europe (Gobiet et al. 2014; Haslinger et al. 2016; Heinrich et al. 2013).

Second, Austrian agriculture is highly diverse and provides multiple services such as high quality food, fodder, fiber and bioenergy, soil and water quality regulation, carbon sequestration, and landscape esthetics. These multiple services are of relevance for other economic sectors such as energy, water, and tourism indicating potential cross-sectoral impacts of climate change adaptation in agriculture.

Third, scientific knowledge on farmers’ perspectives on climate change adaptation is limited to a small region and selected adaptation measures (Pröbstl-Haider et al. 2016). It lacks on a profound understanding of Austrian farmers’ climate change and adaptation appraisal even though it has been argued that such information could be useful to inform adaptation engagement strategies, outreach efforts, and the design of agricultural and climate policies (Arbuckle et al. 2014). Even more, practitioners working in agricultural and environmental organizations, i.e. agricultural knowledge brokers, perceive that Austrian farmers’ adaptation efforts are limited and climate change-related challenges and chances are not fully addressed in different Austrian farming regions (Mitter et al. 2018). The farmers’ lack in timely adaptation is evident even though selected adaptation measures have been identified effective for particular farming regions (Kirchner et al. 2015; Mitter et al. 2015; Schönhart et al. 2014), and collaboration between scientists and agricultural stakeholders has been intensified in the context of climate change adaptation (see e.g. Mitter et al. 2014).

We aim to explore perceptual and socio-cognitive processes influencing Austrian farmers’ climate change and adaptation appraisal, which are expected to create either agricultural adaptation or avoidance. In our analysis, we consider both response strategies, while previous studies have focused on adaptation intention and mostly ignored avoidance (except for Kuruppu and Liverman 2011). In particular, we investigate how farmers’ adaptation intentions and avoidance are formed by considering the following research questions: (i) How do Austrian farmers perceive and appraise climate change? (ii) To what extent are Austrian farmers aware of climate change adaptation measures and how do they appraise their scope of applicability? (iii) How do Austrian farmers’ adaptation intentions develop and how do socio-environmental context factors matter?

Despite purposeful selection of farming regions is standard for this type of research (Woods et al. 2017), it also means that the research results for Austrian farmers are of limited transferability to other cultural contexts and geographic regions. However, the results may be partially translated to farming regions that share similar cultural values and norms and are located in comparable agro-environmental zones, i.e. continental and alpine south as reported in Metzger et al. 2005. Accordingly, we may expect similar response strategies to climate change, i.e. adaptation intention or avoidance, in several farming regions in Central and Eastern Europe, where investigations on farmers’ climate change perceptions and adaptation intentions are limited.

The article is structured as follows. First, we provide a review on theories relevant for analyzing perceptual and socio-cognitive processes in agricultural adaptation. Then, we summarize data collection and analysis, and present empirical findings with respect to climate change and adaptation appraisal, agricultural adaptation intention, and avoidance. Finally, we discuss our empirical findings and draw conclusions.

## Analyzing Perceptual and Socio-Cognitive Processes in Agricultural Adaptation

A variety of recent analyses has investigated farmers’ perceptions of climate change and their intended adaptation behaviour. They differ in theoretical assumptions and identified drivers for agricultural adaptation, which are reviewed and summarized in the following. Reviewing theories relevant to agricultural adaptation intentions highlights their complementarities and summarizing results from their application in various farming regions emphasizes their usefulness for understanding causes of intended behaviour. Based on the review results, we have chosen a theoretical model for our empirical analysis, which is presented below.

### Review of Theories Relevant for Climate Change Adaptation in Agriculture

Behavioural theories explain adaptation intentions through various perceptual and socio-cognitive processes. Of particular relevance to agricultural adaptation are (i) the Theory of Planned Behaviour (TPB; Ajzen 1985) that draws on the Theory of Reasoned Action (TRA; Fishbein and Ajzen 1975), (ii) the Values Beliefs Norms Theory (VBN; Stern 2000), (iii) the Protection Motivation Theory (PMT; Rogers 1983; Rogers and Prentice-Dunn 1997), and (iv) the Model of Private Proactive Adaptation to Climate Change (MPPACC; Grothmann 2005; Grothmann and Patt 2005), which has its roots in PMT.

TRA and its extension, the TPB, are based on proximal causes of an individuals’ particular behaviour and focus on intra- and interpersonal factors such as ‘attitude toward the behavior’ and ‘subjective norm’ (Ajzen 1985; Fishbein and Ajzen 1975). Compared to TRA, TPB incorporates ‘perceived behavioural control’ as an additional factor, which refers to the person’s perception of personal control over the outcome of a particular behaviour. TRA and TPB have proven useful for explaining how the mentioned factors affect intended behaviour. However, they do not provide insights into other relevant intrapersonal factors such as habits, emotions or experience and do not reflect on socio-environmental context factors. This is also true for VBN, which emphasizes normative influences on intended behaviour and pays limited attention to socio-environmental context factors. VBN suggests that personal values are antecedents of environmental beliefs, and personal norms are activated when a person believes that their violation would adversely affect things he or she values. Together, personal values, beliefs (including environmental worldviews, understanding causes and effects of environmental threats, and personal capacity to address these threats), and moral norms explain a considerable amount of variance in pro-environmental behaviour (Stern 2000). Similarly, PMT assumes that the perception of threat and the desire to avoid damage motivates people to change their behaviour or protect themselves (Floyd et al. 2000). PMT is organized along the two cognitive processes of ‘threat’ and ‘coping appraisal’, whereas socio-environmental context factors are not considered. In their MPPACC, Grothmann and Patt 2005 reframe the two cognitive processes of PMT to ‘climate change risk’ and ‘adaptation appraisal’ and extend the model by socio-environmental context factors.

Although TPB, VBN, and MPPACC share two major components, i.e. perceptual and cognitive processes that stimulate and guide behaviour as well as the perceived personal capacity to engage in risk reduction (McLeod et al. 2015) the reviewed theories differ in their focus and consideration of intrapersonal and socio-environmental context factors. Perceptual and cognitive processes that stimulate and guide behaviour refer to behavioural, normative and outcome beliefs in TPB, to personal values and beliefs (i.e. ecological worldview paradigm) in VBN, and to climate change risk appraisal (i.e. perceived probability and severity) in MPPACC. The perceived personal capacity to engage in risk reduction refers to perceived behavioural control in TPB, to perceived ability to reduce threat in VBN, and to perceived self-efficacy in MPPACC. In contrast to the other theories explained above, only the MPPACC introduces context factors with particular relevance for climate change adaptation.

### Major Drivers of Agricultural Adaptation in Different Cultural and Geographic Contexts

The reviewed theories and models have been empirically tested, modified, or refined for the agricultural adaptation context at different locations. For instance, Roesch-McNally et al. 2017 combine TRA and TPB with the ‘anatomy of adaptation’ suggested by Smit et al. 2000 to build a new conceptual framework for investigating adaptation intentions under a climate change scenario. They show that U.S. Corn Belt farmers intend to implement particular adaptation measures if they believe in their efficacy which farmers may learn from own experience or experience of their peers. Niles et al. 2016 apply TPB in two farming regions in New Zealand and find that intended and actual implementation of adaptation measures are influenced by different factors. They highlight that climate change attitudes and beliefs are only related to intended but not to actual adaptation. Arunrat et al. 2017 use TPB and show that adaptation intention of farmers in a Thai region is mostly affected by perceived behavioural control factors, followed by attitude and subjective norm. Behavioural control is operationalized by assessing farmers’ self-confidence in adjusting current farming practices in the next three years, attitude refers to perceived importance and usefulness of adaptation, and subjective norm relates to perceived social pressure on adaptation behaviour. Hu et al. 2006 use TPB to examine farmers’ use of weather and climate forecasts in eastern Nebraska and the U.S. Corn Belt. Their results suggest that farmers perceive the usefulness of forecasts as rather low and depend on expert knowledge for a regular use.

Research on farmers in the U.S. state of Iowa is based on the VBN and reveals that farmers’ belief in climate change significantly influences perceived risks from climate change, which, in turn, fosters agricultural adaptation. Their findings also suggest that farmers who believe in human induced climate change are more likely to support public action on greenhouse gas emissions. However, causal attribution seems to be less relevant for decision-making in agricultural adaptation (Arbuckle et al. 2015). Sanderson and Curtis 2016 operationalize VBN and demonstrate the importance of cultural factors for farmers’ climate change risk perception, which informs their decisions to apply adaptive water conservation strategies in an Australian region.

Duinen et al. 2014 base their investigation on PMT and find that Dutch farmers’ motivation to adapt to droughts can be explained to a significant extent by cognitive factors, i.e. perceived severity of potential consequences, perceived control efficacy, adaptation cost and self-efficacy. McClaran et al. 2015 use PMT to structure their analysis on livestock ranchers in the U.S. state of Arizona. Their findings suggest that the perceived efficacy of selected drought adaptation measures is positively related with implementation. Dang et al. 2014 draw on PMT to explore farmers’ perceived risks and adaptation intentions in the Mekong Delta, Vietnam, and find that risk perception increases with belief in climate change, experienced impacts and lack of public adaptation programs. Building upon PMT, Truelove et al. 2015 analyse paddy farmers’ drought adaptation intentions in Sri Lanka and conclude that efficacy beliefs are the most substantial predictor.

Kuruppu and Liverman 2011 adopt elements from MPPACC for the small island of Kiribati and reveal that belief in self-efficacy drive intentions to adapt to water stress. Similarly, Burnham and Ma 2017 use MPPACC and find that perceived self-efficacy including human and financial capital, institutions and entitlements as well as information, technology and other infrastructure increases adaptation intention among small-holder farmers in China. Woods et al. 2017 build on MPPACC and find a positive correlation between Danish famers’ concern about climate change and intended adaptation to negative impacts. Their results also suggest that farmers respond to potential opportunities emerging from climate change. Frank et al. 2011 incorporate social identity, which represents an individual’s sense or knowledge of membership in a social group, in MPPACC and confirm its importance for the formation of adaptation intentions in an empirical analysis of coffee farmers in Chiapas, Mexico. Consistent with MPPACC, Eakin et al. 2016 explore the effects of objective and subjective capacities on farmers’ adaptive capacity in central Arizona, U.S. and suggest that ‘linking’ capacities such as political and social capital are required for collective action.

The summarized empirical results imply that pertinent adaptation measures, prevailing farming systems, cultural values, and norms as well as geographic regions are contexts that need to be considered for operationalizing theories and framing research questions.

### Theoretical Model for the Empirical Analysis

**Fig. 1:**
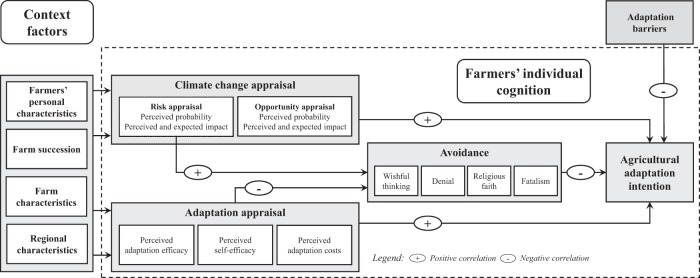
Theoretical model for analyzing farmers’ individual perceptions and cognition towards climate change and adaptation based on the MPPACC by Grothmann and Patt 2005

We have chosen the theoretical MPPACC as heuristic for our empirical research, because it is well suited for structuring farmers’ narratives and analyzing farmers’ climate change and adaptation appraisal as well as their effects on adaptation intention and avoidance including socio-environmental context factors. Building on MPPACC, we empirically examine farmers’ individual perceptions towards climate change and adaptation and consider how socio-environmental context factors affect adaptation intention (see Fig. 1). We assume that an individual has to perceive and evaluate a certain risk or opportunity in a first step (climate change appraisal), and evaluate his or her subjective capability to deal with this risk or opportunity in a second step (adaptation appraisal). While MPPACC is limited to risks and explicitly excludes opportunities (Grothmann and Patt 2005), we consider both risks and opportunities following the commonly accepted definition of climate change adaptation (IPCC 2014). Adaptation appraisal consists of three sub-components, i.e. ‘perceived adaptation efficacy’, ‘perceived self-efficacy’, and ‘perceived adaptation costs’, which are closely related and thus added up.

Depending on the outcome of the two steps, we consider two socio-cognitive pathways an individual may follow to deal with climate change, i.e. agricultural adaptation intention or avoidance. Agricultural adaptation refers to measures that prevent physical or monetary damage or provide new opportunities, whereas avoidance relates to reactions such as ‘wishful thinking’, ‘denial’, ‘religious faith’, and ‘fatalism’ that typically prevent negative emotional consequences of perceived risks and expected impacts. Climate change appraisal (including risk and opportunity appraisal) and adaptation appraisal (including perceived adaptation efficacy, self-efficacy, and adaptation costs) are assumed to be positively related to adaptation intention (as indicated by the ‘+’ sign in Fig. 1). Risk appraisal is assumed to be positively (+) and adaptation appraisal is assumed to be negatively related (−) to avoidance indicating that an individual would respond with ‘avoidance’ if he or she appraises climate change as a risk but is not aware of adaptation measures that are effective, practicable and efficient.

According to MPPACC, adaptation intention is the necessary prerequisite for actual adoption of adaptation measures, while avoidance is discerned as a barrier to adaptation (Grothmann and Patt 2005). Additionally and similar to MPPACC, socio-environmental context factors such as farmers’ personal characteristics, farm characteristics including farm succession, regional characteristics, adaptation barriers as well as an individual’s perception thereof are considered. With respect to farmers’ personal characteristics, we follow the classification by Schmitzberger et al. 2005 and consider farmers’ state of knowledge, their age as well as their attitude towards agriculture and the environment. Farm characteristics include farm size, farm type, cultivation system, major production activities, and future perspectives (i.e. farm succession). Criteria related to regional characteristics comprise freshwater availability as well as land cover, land use, and landscape patterns.

## Material and Methods

Drawing on MPPACC, we have employed a qualitative interview approach for investigating farmers’ climate change and adaptation appraisals, adaptation intentions, and avoidance. The qualitative interview approach allows us to examine how farmers’ mental conditions, their adaptation actions and socio-environmental context factors are interlinked. Furthermore, it enables us to better understand farmers’ social construction of reality and analyze individuals’ cognition, subjective views and social realities, including beliefs, attitudes, and perceptions. Its major strength is to identify patterns in the formation process of adaptation intentions that exist in the Austrian agrarian society. These patterns may correspond to the assumptions of the theoretical model but may also go beyond.

We aimed at interviewing a diverse group of farmers from two major farming regions in Austria, the Mostviertel and South-East Styria, in order to record a broad spectrum of farmers’ climate change and impact perceptions as well as adaptation intentions. We applied the technique of maximal variation sampling where the sample size is typically small but maximizes the diversity relevant to the research question (Flick 2009). The farmers’ and the farms’ diversity was considered with respect to gender and age, major production activities, chosen cultivation system, farm size, full/part-time farming, and topographic and pedo-climatic heterogeneities of the location (see SM 4 in the supplementary material).

We used direct and indirect approaches for getting in contact with potential interviewees. Direct approaches imply that scientists identify potential interviewees contributing to the maximal variation sample directly, e.g. via online search or established professional or personal networks. Indirect approaches mean that potential interviewees are suggested by a ‘gatekeeper’ such as extension experts affiliated to the regional chambers of agriculture or through snowball sampling where already interviewed farmers provide new contacts (Helfferich 2005). Most interviewees were recruited indirectly, i.e. via extension experts who provided farmers’ contact details, and directly through the interviewer’s network (see SM 4). We aimed at conducting the interviews with the person(s) responsible for adaptation decision-making. Depending on the decision competences on the farms, we included farm managers and possibly other family members. In total, twenty semi-structured face-to-face interviews were conducted with 29 persons. Thirteen interviews were held with a single farm manager and another four interviews with a farming couple. One interview took place with a farmer and his successor and one with a farming couple and their successor. Another interview involved three generations, i.e. a farmer and his daughter and grandson. Participation in the interview was voluntary and the interviewees decided on the number of participants. Involving all persons responsible for adaptation decision-making on a farm in an interview is reasonable because such an approach stimulates the interviewees, supports them in recalling activities and events, provides some quality control, and can lead beyond the responses of individuals (Flick 2009; Patton 2002).

The face-to face interviews were carried out on the farms of the interviewees during the growing period in 2016. An interview guide with open-ended questions was designed (see SM 1) covering the concepts of the theoretical model introduced above. Open-ended questions were asked in order to encourage farmers to report more rather than less, to gain farmers’ personal information, and to elicit new phenomena and unexpected structures, as recommended by Lamnek 2010 and Weischer 2007. The interview guide supported the interviewer in tailoring the questions to the interviewees and in ensuring that all relevant aspects were covered. It was pre-tested with farmers who were not included in the original sample and slightly changed based on the received feedback. Each interview started with the introductory question: “*To start with, would you please share your view on climate change?”* with the intention of stimulating narration, and obtaining information on farmers’ belief in and attitude towards climate change. Further topical areas of the interview guide were (i) perceived and expected changes in climate conditions, (ii) perceived and expected climate change-related impacts, (iii) adaptation measures already implemented or planned to be implemented in the future, and (iv) factors stimulating or impairing the implementation of adaptation measures. The sequence of the questions and their particular wording were adjusted to the interview situation in order to keep up the ‘narrative line’ (Weller et al. 2010).

At the end of the semi-structured interviews, which lasted between 30 and 120 min the interviewees were asked to fill out a standardized one-page questionnaire about demographic data, i.e. year of birth, gender, and farm structural data, i.e. major production activity, cultivation system, farm size, and full/part-time farming (see SM 2). After the interview, the interviewer took structured notes to summarize interview atmosphere and context (see SM 3).

According to the criterion of ‘theoretical saturation’ (Glaser and Strauss 1967), sampling was stopped when informational saturation was reached, i.e. when no new insights were obtained. The sample size is sufficient because of the explorative character of our study which aims at describing a detailed picture of Austrian farmers’ perceptions and adaptation intentions in various farming contexts and because of combining the maximum variation sample and the saturation criterion (Baker and Edwards 2012; Crouch and McKenzie 2006).

Each farmer agreed to a confidential use of the word-for-word transcript of the interview by signing a consent form. The farmers were given the opportunity to correct factual errors in the interview transcript before data were further analyzed. The interviewees were coded in the transcripts in order to guarantee confidentiality. The codes describe the farming regions (M for Mostviertel, and S for South-East Styria), the interview held in the region numbered consecutively from 1 to 10, and—in case of multiple interviewees in one interview—an additional number referring to the interviewee (e.g. M4,1 and M4,2; see SM 4). These codes are used in the results section to trace the interviews.

The interview transcripts were analysed systematically by means of a qualitative content analysis (see Mayring 2010, Kuckartz 2014). Facilitated by the software Atlas.ti (see atlasti.com for more information), the qualitative content analysis was performed in two steps. In the first step, codes were deductively defined based on the theoretical model outlined above, and relevant text passages of the empirical material were assigned to these codes in Atlas.ti. Deductive codes included, for example, belief in climate change, perceived/expected changes in climate conditions, perceived/expected climate change-related risks/opportunities, already implemented/planned adaptation measures, perceived effectiveness/efficiency of adaptation measures, and perceived adaptation costs. Code definitions were based on the relevant theoretical literature (Grothmann 2005; Grothmann and Patt 2005; Grothmann and Reusswig 2006) and saved as code comments in Atlas.ti. In a second step, these codes were further decomposed inductively, i.e. based on the empirical material, for instance to introduce sub-categories or an evaluation. Furthermore, new codes were added for emerging topics. The combination of theoretically- and empirically-driven coding is known as deductive-inductive strategy and widely recommended in the literature in order to increase analytical flexibility and thus utilize the qualitative material as effectively as possible (Gläser and Laudel 2013; Kuckartz 2014; Schreier 2014). It should be noted that there is no algorithm for automatically assigning text passages of the interview transcripts to the defined codes. Developing and applying the code system is an interpretative process based on the intellectual performance of the analysts. The code system was developed in close cooperation by a team of researchers, whereby text segments of the interview transcripts and respective coding were discussed thoroughly in order to improve and refine the code system iteratively.

## Results

In the following, we suggest pathways through the MPPACC by presenting four different groups of farmers and how adaptation intention and avoidance are formed within these groups. Furthermore, we link the relevant context factors to the identified groups of farmers. The results are summarized in Table 1.

**Table 1:** Summary on the major components of the Model of Private Proactive Adaptation to Climate Change (MPPACC) for the four, empirically-based groups of farmers, which differ in the formation process of adaptation intention and avoidance

Components of the MPPACC	Climate change adaptors	Integrative adaptors	Cost-benefit calculators	Climate change fatalists
Climate change appraisal (Risk and opportunity appraisal)	Belief in global CC Risk perception dominates Negative CC impacts experienced and expected in the future	Skeptical about global CC Risk and opportunity perception Negative impacts attributed not only to CC but also to other causes Ambiguous expectations about future climate conditions	Skeptical about global CC Risk and opportunity perception Negative impacts attributed not only to CC but also to other causes Expect marginal negative CC impacts	Belief in global CC High risk perception and highly negative feelings about CC and related impacts Few negative CC related experiences Future of agriculture appraised extraordinary difficult
Adaptation appraisal (Perceived adaptation efficacy, self-efficacy, adaptation costs)	Awareness of effective and efficient AM Successful implementation of AM High self-responsibility for their farms AC perceived as high but necessary for future success	Environmental-friendly farming practices appraised as effective and efficient AM and successfully implemented CC of limited importance for implementation of AM High self-responsibility for their farms AC only mentioned in the context of savings	Awareness of effective AM Successful implementation of selected AM High self-responsibility for their farms AC perceived mostly too high compared to expected benefits	No awareness of effective and efficient AM Responsibility delegated to the government, the consumers, and a potential farm successor Financial capacity to implement AM perceived low
Avoidance	Not addressed	Denial, wishful thinking	Wishful thinking, superstition, unrealistic optimism	Fatalism, resignation
Personal characteristics	Comprehensive knowledge on CC and AM Societal engagement	Pro-environmental attitude Desire for independence	Comprehensive knowledge on AM Market orientation Desire to run farm economically viable and crisis-proof Young	Conservative Trust in habits and traditions Poor skills in problem-solving and innovation
Farm characteristics	Heterogeneous	Mostly full-time farmers Mostly organically certified	Mostly full-time farmers Heterogeneous farm types	Specialized farms, i.e. no diversification Farm size above the Austrian average but farmers perceive their farms as small
Farm succession	Determined	Not mentioned	Determined	Not determined
Regional characteristics	Freshwater availability, LC, LU, and landscape patterns	LC, LU, and landscape patterns	LC, LU, and landscape patterns	Not perceived relevant
Adaptation intention	Yes	Yes	Yes, with a long-term planning horizon, i.e. if AC are lower than expected benefits	No

*AC* adaptation costs, *AM* climate change adaptation measures, *CC* climate change, *LC* land cover, *LU* land use

### Climate Change Adaptors

The first group of farmers, the ‘climate change adaptors’, perceive climate change mostly as a risk, have successfully implemented adaptation measures on their farms, indicate adaptation intentions and do not address avoidance.

In the context of **climate change appraisal**, the climate change adaptors report a variety of perceived changes in regional climate conditions and attribute their perceptions to climate change. Perceived changes include shorter and milder winters with little or no snow, an increase in the number of sunny days and sun intensity, a higher frequency of summer droughts as well as an increase in ‘chaotic’ and hardly predictable weather conditions such as early onset of spring and snow in late spring. The climate change adaptors have already experienced a bandwidth of negative impacts and expect that these impacts will affect their farms in the future as well. Accordingly, risk perception dominates and perceived opportunities related to climate change are rare. In particular, the farmers report decreasing yield levels and crop failure, pest pressure and sunburn in orchards as well as pest pressure, wind and snow breakage in forests. They also address regional water shortages as a potential future risk. Even though the farmers appraise the prolongation of the growing period positively they highlight the risk of late frosts in this context. Furthermore, they see potential yield increases rather as a direct consequence of breeding advances than of more favorable growing conditions. However, the farmers associate opportunities with particular adaptation measures that could gain in importance because of regional warming, e.g. the diversification in crop or fruit choices, as indicated by one farmer:

“And maybe it’s a chance to cultivate other fruits in the medium term. We actually have some test sites, all sorts of things, kaki, figs, kiwi etc. Fruit that we did not even think about 10 to 15 years ago because they did not work. Now they grow and not bad at all.” (S7)

The climate change adaptors **appraise adaptation** on their farms positively. They know effective adaptation measures and have already implemented selected measures on their farms. Prominently discussed examples are humus formation activities, and investment in technologies such as irrigation or hail protection nets. The climate change adaptors explicitly aim at adapting their farms to climate change, they are confident of their capability to reduce the expected negative impacts and perceive themselves responsible for their farms. As such, short- and long-term decision-making is influenced considerably by climate change. The farmers also address adaptation costs, which they consider as high but necessary for successful adaptation.

**Adaptation intentions** are expressed in the context of planned or intended adaptation measures. The farmers mention to continue their efforts devoted to humus formation, to further invest in technologies and expand their field of application, e.g. irrigation applied during dry spells and for frost protection, to convert to organic farming or to change their major production activity, e.g. through diversification of farm income or changing from fruit to wine production.

**Context factors** can be classified into farmers’ personal characteristics, farm characteristics including farm succession, and regional characteristics. Relevant **personal characteristics** of the climate change adaptors’ comprise their state of knowledge, preferred information sources, and societal engagement. One farmer officially representing fruit growers feels responsible to establish long-term adaptation measures beyond his own farm:

“I ask politics to keep the next generations in mind. According to the scientists, our region is threatened to turn into steppe … because the influence of the Pannonian lowlands will increase. Our region will experience repeated heavy rain falls and long periods without any rain. If we want to maintain agricultural production in our region, we need to make sure that we have water. My goal is to promote a project for the next generations.” (S5)

**The farmers** of this group are well informed and know a lot about climate change and agricultural adaptation. Their preferred information sources are print media such as agricultural magazines, weather records on their farms, and personal exchange with scientists. They rated their information sources as reliable, and also gave credence to alarming information. Some of the farmers are politically active and engage in climate change adaptation beyond their farms. **The farms** managed by climate change adaptors are heterogeneous. They are run as full or part-time farms and focus on various production activities. However, farmers of this group mentioned that climate change impacts depend on soil conditions and the location of their fields. For instance, waterlogging on heavy soils is considered challenging for elder production and fields scattered over the farming region are perceived to reduce damages caused by typically local events such as hail or thunderstorms. **Farm succession** is determined on all the farms, i.e. the farm successor is either involved in decision-making or is already responsible for the management of the farm. **Regional characteristics** that climate change adaptors perceive relevant for agricultural adaptation are freshwater availability, e.g. for implementing large-scale irrigation as well as land cover, land use, and landscape patterns because of their effect on development cycles of pests.

The climate change adaptors do not perceive **barriers specific to agricultural adaptation**. They rather mention challenges in the more general context of farm development and refer to low prices of agricultural commodities, high workload in a small-scaled environment, and high labor costs for seasonal workers. They would appreciate higher payments from the national disaster fund and reduced social security premiums in years of massive losses as well as additional insurance products to cushion the impacts of extreme weather events.

### Integrative Adaptors

The second group of farmers, the ‘integrative adaptors’, continuously adapt to changes—including changes in regional climate conditions—and intend to do so in the future. Adaptation is seen as part of a holistic lifestyle including an environmental-friendly farming practice or as a possibility to improve the resilience of the farm and take advantage of potential emerging opportunities. Some of the farmers also express avoidance, which becomes apparent through denial or religious faith.

The integrative adaptors address **changes in regional climate conditions** such as a gradual temperature increase, a slow ‘disappearance’ of transition periods between seasons, warmer winters with little snow, and more frequent or severe heavy rainfall events. However, they express uncertainty about the relationship between perceived regional climate conditions, global climate change and related impacts by referring to their own experience, which results in ambiguous expectations about future climate conditions. The integrative adaptors frame their observations and perceptions as natural climate variation and refer to human influence as additional driving factor. The following quotes illustrate the equivocal opinion of the farmers forming this group:

“I think, climate change has always existed, as far as we know from history […]. Climate change is happening – caused by nature or, I don’t know, whoever, whatever are causes. But I think humans contribute to it, such that it has reached this speed. […]. But there has always been climate change – we know that. But which factors are related is certainly the big question.” (M10)

“We had harsh winters in the past, and we had winters with only little [snow]. We had hot summers in the past … there were wet summers, dry summers, we had all that in the past. So, with my 60 years, I cannot evaluate if anything has changed.” (M2)

With respect to **climate change appraisal**, the integrative adaptors report risks and opportunities. In terms of risks, they refer to lower yield levels in crop and grassland production, increasing weed and disease pressure, and windfalls in forests. However, farmers attribute experienced negative impacts only partly to variations in regional climate conditions and add other potential causes. For instance, the farmers perceive negative impacts of floods and soil water erosion not only as a result of more frequent or severe heavy rainfall events, but highlight more intensive maize cultivation and rising shares of building land as important causes. Weed and disease pressure are not only attributed to changes in their development cycles but are mostly seen as a consequence of organic production with limited pesticide application. The farmers also reflect on opportunities in the context of climate change. They refer to increasing temperature levels leading to a prolongation of the growing period and the grazing year for livestock.

In the context of **adaptation appraisal**, the integrative adaptors emphasize that the application of environmental-friendly farming practices, which are adjusted to a farm’s resource base is perfectly adequate for climate change adaptation as well. In particular, the farmers refer to a focus on soil health, humus formation, cultivation of cover crops, the diversification in production activities, and organic production. These adaptation measures are evaluated positively for both, the environment and climate change adaptation. However, the farmers mention strong personal responsibility for natural resources, the environment, God’s creation, and the future generations as well as the desire for economic independence as major stimuli for action whereas climate change is perceived of limited importance. Adaptation costs are only mentioned in the context of savings resulting from circular economy.

**Adaptation intention** is expressed as continuation or expansion of environmental-friendly or organic production, which is perceived as the basis for taking advantage of new opportunities. However, the fundamental belief in the effectiveness of the chosen farming practices and production methods for climate change adaptation may lead to overconfidence and thus avoidance. Expressions of **avoidance** can be interpreted as religious faith or denial (gallows humor) as illustrated by the following example:

“We won’t be affected very much if temperature increases by two degrees or more. Unless all glaciers melt and the water comes and stays, as floodplain or as a lake for swimming. … In that case, we will build a wine tavern over there (laughs).” (M2)

Perceived **context factors** are summarized as personal, farm, and regional characteristics. The **integrative adaptors** are characterized by their deep appreciation of nature, their high environmental awareness, and their desire for independence. This results in a high environmental or sustainability performance of **their farms**. The farms are organically certified except in one case. This farmer follows the guidelines for organic production but does not want to forgo the use of chemical pesticides in extreme years. Environmental-friendly production is perceived to decrease the farm’s vulnerability to regional climate change but to increase its susceptibility to pest pressure. Full-time farming is predominant in this group but part-time farming also occurs. With respect to **regional characteristics**, integrative adaptors refer to land cover, land use, and landscape patterns because of their effect on soil water erosion and nutrient emissions.

The integrative adaptors do not perceive **adaptation barriers** for their farms. However, they are concerned about general developments in the agricultural sector that may decrease the adaptive capacity of the farms. The farmers mention, for instance, low societal appreciation for organic production and thus low prices for agricultural commodities, inflexible regulations and high administrative burden, untargeted subsidies, highly industrialized processes along the agricultural value chain, high emissions, and exploitation of natural resources.

### Cost-Benefit Calculators

The third group of farmers, the ‘cost-benefit calculators’, observe changes in regional climate conditions but expect marginal climate change impacts. Nevertheless, they are aware of effective adaptation measures and have already implemented adaptation measures that bring about slight or moderate changes on their farms. They intend to implement more elaborate adaptation measures that change the farms’ characteristics or strategic orientation solely if damages exceed a certain level, i.e. are higher than adaptation costs. Some of the cost-benefit calculators also articulate avoidance, mostly through wishful thinking.

With respect to **climate change appraisal**, the cost-benefit calculators think that climate change is happening but they are skeptical about its extent at regional level and related risks for their farms. They argue that climate change is not new but has occurred throughout global history because they perceive only slight changes in regional climate conditions that are ‘beyond natural variation’. Based on their experiences, they assess information on climate change and potential regional impacts provided by mass media as unreliable and exaggerated.

“Okay, my opinion on climate change – I think there have always been changes in the climate but the media reports are a bit over the top nowadays. Well, it’s because some want to do business with it.” (M6)

Nevertheless, the cost-benefit calculators perceive changes in regional climate conditions and address ‘global warming’ by referring to increasing mean temperatures, mild winters with little snow, and the prolongation of the growing period. Some farmers mention an increase in extreme weather events such as late frosts and heavy rainfall. However, the cost-benefit calculators assess the climate-related risks for their farms as low even though they have already experienced negative impacts such as yield reductions due to extreme weather events and high pest pressure in forests resulting from higher temperatures. They ascribe their low risk perception to regional characteristics, farm-specific preconditions, and appropriate farming practices. For instance, they mention soil water erosion in the context of heavy rainfall but relate their low vulnerability to favourable soil conditions, mostly flat topography or conservation tillage. They also refer to other off-farm developments such as soil compaction and sealing that contribute to an increase in soil water erosion. Considering opportunities, the cost-benefit calculators mention an increase in yield level and quality because of higher temperatures as well as relaxed autumn fieldwork because of a prolonged growing period. For the future, the farmers expect rather slight changes in regional climate conditions leading to the prolongation of the growing period, reduced water availability during the growing period, and an increase in pest pressure.

“Maybe it will be a bit warmer. But I think that temperature will level off. I do not assess this tragically. […] I think I have to react in case that my current farming practices are not working anymore. If that [climate change] remains at its current level, then we will be able to handle it, somehow.” (S8)

The ability to adjust farming practices to upcoming challenges in an economically efficient way is seen as a core competence of a farm manager. According to this self-conception, cost-benefit calculators do not regard climate change as an outstanding challenge, but as a ‘regular’ management duty. Consequently, they continuously implement adaptation measures such as changes in crops and varieties and conservation tillage even though they do not consider them as climate change adaptation. With respect to **climate change adaptation appraisal**, the cost-benefit calculators consider themselves responsible for their farm and the timely implementation of adaptation measures. They are aware of effective adaptation measures that change the farms’ characteristics or strategic orientation such as extending vineyards over cropland and investment in new technologies. However, the adaptation costs of adaptation are currently perceived too high, compared to expected benefits.

When climate change adaptation is explicitly addressed, the cost-benefit calculators form **adaptation intention** with a long-term planning horizon. The farmers argue that they generally base their farm management decisions on cost-benefit analyses. Therefore, they balance perceived risks related to climate change against adaptation costs, which delays actual implementation of more elaborate adaptation measures that change the farms’ characteristics or strategic orientation.

The cost-benefit calculators stress the economic paradigm as the basis for decision-making. Nevertheless, the interviews reveal some irrational elements, as illustrated by a farmer’s quote indicating that he rather believes in superstition than accepting and appraising the severe climate change-related damages, which he had experienced during the year of data collection.

“This year is a leap year. It is a leap year and a ‘Friday-year’ because it began on a Friday. In earlier days, people have always said that leap years and Friday-years are somewhat extreme. Believing in these sayings helps.” (S1)

Others express **avoidance** through wishful thinking, i.e. they expect adverse climate change impacts in the future but belief to remain unaffected despite of other farmers.

**The cost-benefit calculators’** major stimuli for adaptation action are economic, legal and policy conditions, farm characteristics (e.g. soil quality, slope, field size), personal interest (e.g. interest in field trials and new crops), and the desire to run the farm economically viable and crisis-proof. Continuous adjustments in farming practices belong to the farmers’ personal identity. It is also notable that all farm managers of the sample below the age of 40 (typically referred to as ‘young farmers’) belong to the group of cost-benefit calculators. **The farms** managed by cost-benefit calculators differ in size and major production activity. They are the main source of income for the cost-benefit calculators—except for one farm specialized in direct marketing, which is run part-time. Furthermore, the farmers report of differences in soil conditions and topography, which affect the severity of climate change impacts. For instance, deep and loamy soils are considered to decrease negative impacts of dry spells and droughts and high altitudes are considered reduce heat stress for plants and livestock. **Farm succession** is either determined or not an actual topic because of the young age of the farm managers. **Regional characteristics** that cost-benefit calculators perceive relevant for expected risks are land cover, land use, and landscape patterns because of their effect on soil water erosion.

The cost-benefit calculators refer to adaptation costs as major **barrier** for implementing elaborate adaptation measures that change the farms’ characteristics or strategic orientation but do not perceive any additional barriers.

### Climate Change Fatalists

The fourth group of farmers, the ‘climate change fatalists’, express highly negative feelings about climate change, which are based on only few adverse experiences. They are not aware of effective and efficient adaptation measures, perceive the government and consumers as responsible for action and thus do not form adaptation intention. The farmers also express avoidance, mostly through fatalism.

Farmers forming part of this group perceive a higher frequency of extreme weather events, rapid changes in weather conditions (warm/cold, dry/wet), and warmer winters. They articulate strongly negative feelings about **changes in regional climate conditions and related impacts** even though they report only individual years with lower yield levels, higher pest pressure, or aggravated workflows. The climate change fatalists evaluate the future of agriculture as extraordinary difficult. However, they do not differentiate between climate change and other factors influencing future developments of their farms.

The climate change fatalists **appraise adaptation** negatively. They describe their farms as small in size with very low capacity to adapt. More than this, they perceive their farms as globalization victims without chances of long-term survival. The farmers are either not aware of effective adaptation measures, rate established measures as ineffective or too expensive for their farms or perceive the conversion from full- to part-time farming as sole adaptation option for their farm. Furthermore, the climate change fatalists do not perceive self-efficacy and delegate the responsibility to the government, the consumers or to a potential farm successor, as expressed in the following quote:

“We hope to make it until we retire. For the successor – one does not know it yet. They have to know themselves what they want or in which direction, they want to develop.” (M3)

The farmers of this group do not form **adaptation intentions** but express **avoidance**, mostly through fatalism. Risk perception combined with the negation of effective adaptation measures turns into resignation and fatalism. For instance, fatalism was expressed by a farmer who is aware of climate change-related risks and has realized that adaptation would be necessary. However, he does not intend to adapt because he is not aware of any viable adaptation measure. Additionally, he seems to be inflexible and unwilling to implement any measure to modernize or change current farming practices. Resignation and fatalism was expressed as follows.

“What can we change?! We are in the downward spiral. That’s sad, […] we don’t know how to proceed, we don’t know how we could survive as farmers.” (S9)

The **climate change fatalists** can be described as conservative, they trust in habits and traditions, show poor skills in problem-solving and innovation, and feel paralyzed because of the negative development of economic, legal, and policy conditions. The climate change fatalists run specialized **farms** and perceive their farms as small and experienced impacts as highly adverse. However, the size of their farms is above the Austrian average and the perceived impacts are lower than one would expect from statistical reports. This indicates the relevance of subjective realities for climate change appraisal. **Farm succession** is not clear and **regional characteristics** do not seem relevant for the farmers’ risk perception, adaptation intentions, and avoidance.

The climate change fatalists do not refer explicitly to **adaptation barriers** for their farms. However, they perceive a variety of economic, legal, and policy developments as negative for the development of the agricultural sector and their farms. For instance, they criticize the current growth paradigm, rigorous animal welfare acts, administrative barriers, concentration in the agricultural value chain, low prices of agricultural commodities, and increasing demand for industrially produced food.

## Discussion

The discussion is structured according to the concepts of the theoretical model, i.e. how climate change and adaptation appraisal as well as agricultural adaptation intention are formed and how socio-environmental context factors influence adaptation intention.

### The Formation of Climate Change Appraisal

Our analysis shows that all interviewed farmers perceive changes in regional climate conditions even though they do not always link these perceptions to global climate change or find these changes alarming. The identified four groups of farmers differ with respect to their belief in global climate change and its materialization at regional level, e.g. through perceived changes in regional climate conditions and impacts on regional farming. While the climate change adaptors and the climate change fatalists believe in global climate change leading to massive changes in regional climate conditions, the integrative adaptors and the cost-benefit calculators are skeptical and argue that perceived changes are an indication for natural variation in climate.

The interviewed farmers report a broad variety of experienced impacts on their farms, which they interpret as climate change risks or opportunities. The climate change adaptors and the climate change fatalists mainly feel affected by risks, but while the climate change adaptors experienced severe negative impacts in the past, the fatalists did not. The fatalists’ risk appraisal seems to be part of a general negative attitude towards future developments. Although the farmers forming part of the two other groups, i.e. integrative adaptors and cost-benefit calculators, report a bandwidth of negative impacts in the context of changing regional climate conditions, they mention non-climatic factors such as changes in land cover as major reasons and do not appraise them as a serious risk for their farms. The integrative adaptors believe in the resilience of their environmental-friendly farming practices and consciously accept some associated challenges, e.g. with pest control. The cost-benefit calculators are aware of climate change risks but appraise them as ‘regular’ management task and are thus not concerned. Except for the climate change fatalists, the farmers forming part of all other groups also reflect on potential opportunities related to climate change. Most of them address opportunities in the context of increasing mean temperatures and the prolongation of the growing period.

These results suggest that climate change appraisal is stimulated by pre-existing beliefs in climate change, which influence how regional climate conditions are perceived and how experienced and expected risks and opportunities are interpreted. Similar findings have been presented in the empirical literature and are known as ‘motivated reasoning’, i.e. prior belief in or knowledge on climate change influences people’s perceptions (Hart and Nisbet 2012; Howe and Leiserowitz 2013; Myers et al. 2013), and ‘assimilation bias’, i.e. people look for and accept evidence that confirm their individual beliefs but ignore dissenting evidence (Lord et al. 1979; Whitmarsh 2011).

### The Formation of Adaption Appraisal

Adaptation appraisal consists of farmers’ perceptions of adaptation efficacy, self-efficacy, and adaptation costs. With regard to adaptation efficacy, the climate change adaptors, the integrative adaptors and the cost-benefit calculators know a bandwidth of effective climate change adaptation measures and have already implemented several of them on their farms. The climate change fatalists are not aware of adaptation measures and have not implemented any on their farms. While climate change adaptors try to balance a broad mix of adaptation measures, integrative adaptors focus on environmental-friendly farming practices adjusted to a farm’s resource base, which they assess to be adequate for climate change adaptation as well. Cost-benefit calculators are continuously taking adaptation measures that bring about slight or moderate changes on their farms. They are also aware of effective adaptation measures that may change the farms’ characteristics or strategic orientation but consider related benefits too low, compared to the costs. These results are in line with previous empirical analyses, which have revealed that preferred adaptation measures differ between groups of farmers sharing, for instance, climate change belief, attitudes, value systems, and perceived risks (Barnes and Toma 2012; Hyland et al. 2016).

Perceived self-efficacy and the capacity to respond to climate change-related challenges has received growing attention in the empirical literature suggesting its relevance in the agricultural context (e.g. Arbuckle et al. 2014; Duinen et al. 2014). In our empirical data, self-efficacy is expressed as the farmers’ sense of personal responsibility for their farms and their perceived capability to act. Except for the climate change fatalists, the farmers show high levels of self-efficacy paired with a high flexibility to respond to changes. While the climate change adaptors and the cost-benefit calculators additionally show a high level of knowledge on climate change, related impacts and potential adaptation measures, perceived self-efficacy of the integrative adaptors is embedded into an environmental-friendly value system. Furthermore, it is notable that farmers belonging to the group of climate change adaptors report personal political engagement in order to increase peoples’ climate change awareness and to evoke adaptation beyond their farms.

The four groups of farmers address adaptation costs differently. While the climate change adaptors consciously accept high costs for successful adaptation, the integrative adaptors emphasize potential savings from measures that yield benefits for both the environmental and adaptation performance of their farms, and the climate change fatalists appraise costs for adaptation measures as too high. The cost-benefit calculators balance expected benefits against adaptation costs, and highlight adaptation costs as major determining factor for implementing long-term adaptation measures. Park et al. 2012 have come to a similar conclusion showing that adaptation costs are particularly decisive for long-term, transformative adaptation.

### The Formation of Agricultural Adaptation Intention and Avoidance

The formation of agricultural adaptation intention and avoidance is stimulated by the farmers’ individual cognition represented by the components of climate change and adaptation appraisal. Our empirical results indicate that a high level of climate change appraisal does not necessarily evoke agricultural adaptation intention and—vice versa—a low level of climate change appraisal does not automatically lead to avoidance. For example, although climate change adopters and climate change fatalists have experienced negative impacts and feel threatened by climate change risks, only the first intend to adapt. Otherwise, integrative adaptors and cost-benefit calculators appraise climate change risks as low for their farms but show adaptation intention in the short- or long-term.

While experienced as well as expected positive and negative climate change impacts do not always stimulate adaptation intention, we find that farmers’ adaptation appraisal is key for the formation of adaptation intention. In particular, awareness of effective and efficient adaptation measures as well as perceived self-efficacy greatly affect whether farmers intend to adapt. The results demonstrate that the climate change adaptors, the integrative adaptors, and the cost-benefit calculators appraise adaptation positively and form adaptation intention, whereas the climate change fatalists appraise adaptation negatively and solely form avoidance. Other case studies have come to a similar conclusion and highlight the strong positive effect of perceived self-efficacy on adaptation intention (Burnham and Ma 2017; Niles et al. 2016) given that the farmers know adaptation measures with a convincing cost-benefit ratio. This result is also conform to the MPPACC suggesting that the combination of positive climate change and adaptation appraisal result in adaptation intention (Grothmann 2005; Grothmann and Patt 2005).

Avoidance is expressed in terms of wishful thinking, denial, religious faith, and fatalism, in our qualitative data material. In case of wishful thinking, the cost-benefit calculators expect adverse climate change impacts in the future but belief to remain unaffected and thus show avoidance. This belief is typically fostered by rather irrational justifications or paired with ‘unrealistic optimism’ (Weinstein 1980, 1983, 1987) suggesting that adaptation is not yet very relevant. Denial and religious faith is revealed when integrative adaptors doubt to be negatively affected by climate change in the future even though they have already experienced adverse impacts. While wishful thinking, denial, and religious faith degrade the risk of climate change, fatalism requires risk perception. In combination with the lack of knowledge on viable adaptation measures, perceived risk turns into resignation within the group of fatalist farmers. These findings also support the crucial role of adaptation appraisal. While the avoidance strategies of wishful thinking, denial and religious faith are related to risk appraisal and leave room for adaptation intention, fatalism is connected to negative adaptation appraisal and farmers belonging to this group do not form adaptation intention.

### Socio-Environmental Context Factors

The socio-environmental context influences the farmers’ individual cognition and thus agricultural adaptation intention and avoidance. We distinguish between farmers’ personal characteristics, farm characteristics including farm succession, and regional characteristics. Criteria related to the farmers’ personal characteristics include the farmers’ state of knowledge, their age as well as their attitude towards agriculture and the environment. The farmers’ personal characteristics seem to be related to their perceived self-efficacy, which is expressed in terms of their perceived personal responsibility for their farms. In our data material, we find knowledgeable farmers who search actively for detailed information (climate change adaptors), ambitious farmers with pronounced environmental awareness (integrative adaptors), determined mainly young farmers following profit and efficiency principles (cost-benefit calculators), and conservative farmers who have developed strong habits (climate change fatalists). Knowledge, environmental awareness and economic interests seem to inspire self-efficacy and encourage adaptation intention, whereas conservatism seems to inhibit adaptation and induce avoidance. Other studies have also addressed personal characteristics and find that market-oriented farmers that perceive farming as profession or vocation as well as well-educated farmers with a high pro-environmental attitude have a higher adaptation intention than traditionalists (Eggers et al. 2015; Hyland et al. 2016).

Farm characteristics and farm succession are other important contexts for agricultural adaptation. For the integrative adaptors and the cost-benefit calculators, they seem to be related to the farmers’ personal characteristics. The sense of environmental responsibility of integrative adaptors becomes apparent in organically certified farms (except for one farm that follows organic production standards in most years). Full-time farming (except for one farm specialized in direct marketing that is run part-time) reflects the economic orientation of cost-benefit calculators. Organic and full-time farming seem to increase adaptation intention. The farms managed by the climate change adaptors differ in size and major production activity and farm succession is fixed. The availability of a farm successor seems to stimulate adaptation intention and the so-called ‘successor-effect’ (Chiswell 2016) motivates even older farmers to adapt. The farms run by the climate change fatalists are specialized, i.e. no diversification, and farm succession is not determined. These results are similar to the findings from Eggers et al. 2015, which indicate that full-/part-time farming, conventional/organic farming as well as farm succession are factors that influence adaptation decision-making.

Regional characteristics such as water availability, land cover, land use, and landscape patterns have been addressed by all farmers, with exception of the climate change fatalists. This indicates that the farmers’ perception of their environment is important in the context of agricultural adaptation, regardless if the farmers refer to regional characteristics as stimulating or inhibiting factors.

Our results emphasize that not only climate change and adaptation appraisal affect the formation of agricultural adaptation intention and avoidance, but personal, farm and regional characteristics are also of importance as well. This finding supports conceptual and empirical literature proposing that adaptation is often a response to a mix of climatic and non-climatic factors (Berrang-Ford et al. 2011; Knox et al. 2010; McLeman et al. 2008; Mitter et al. 2018; Smit and Skinner 2002). Farmers indicating that economic factors are of major relevance for decision-making and even more important than climatic factors, e.g. the cost-benefit calculators, can be interpreted in the context of the ‘finite pool of worry effect’ (Hansen et al. 2004; Weber 2006, 2010), which suggests that increased concern, e.g. about market development, may result in decreased concern about climate change. It may also be useful to follow the concept of ‘multi-purpose’ and ‘hidden adaptation’ (Grüneis et al. 2016), when referring to measures that are mainly motivated by non-climatic factors but contribute to climate change adaptation as well. For instance and following the argumentation by the integrative adaptors, changes in crop rotations may result from the desire to support humus formation (environmental factor) but may positively affect crop production through changes in the growing period (climatic factor) as well.

## Conclusions

We have explored farmers’ climate change and adaptation appraisal in order to better understand how farmers’ adaptation intentions are formed through perceptual and socio-cognitive processes. The theoretical Model of Private Proactive Adaptation to Climate Change (MPPACC, Grothmann 2005; Grothmann and Patt 2005) served as a basis for conducting semi-structured interviews with 29 farmers in two Austrian farming regions. The interviews were analyzed by means of a qualitative content analysis. The theoretical model and employed methods are transferable to other contexts and regions, which could broaden the thematic and geographic scope of adaptation research and, at the same time, increase comparability between empirical analyses. Our results from the qualitative content analysis are of particular relevance for farmers, farming and adaption experts, agricultural knowledge brokers, and policy and decision-makers in Austria. In the scientific context, they could serve as a basis for deriving items for a standardized survey in a quantitative follow-up study.

Our empirical data suggest that farmers’ belief in and perception of climate change shapes risk and opportunity appraisal, presumably through a process of motivated reasoning. Furthermore, experiences in climate change and related impacts affect climate change appraisal. Doubts about climate change—which were raised by integrative adaptors and cost-benefit calculators—indicate that farmers’ knowledge and information level about climate change may be lower than expected by farming experts and agricultural knowledge brokers, who suppose that farmers are aware of climate change (see e.g. Grüneis et al. 2016; Mitter et al. 2018). The mass media does not seem to stimulate learning about climate change very much because farmers are critical about the validity of the transmitted information. Thus, it appears relevant to evaluate and monitor campaigns for awareness raising in order to improve information and knowledge brokerage.

According to the interview results, trusted information sources play an important role for transferring up-to date and understandable information inducing action within the group of climate change adaptors. Therefore, the regional farmers’ organizations could serve as peer groups for knowledge transfer, which makes knowledge on their attitudes and perceptions towards climate change even more important (see e.g. Mitter et al. 2018). Furthermore, information may not be limited to risk maps and highlights of emerging opportunities, but have to comprise of effective adaptation measures tailored to the farming region and farmers’ needs as well. This is of particular importance because adaptation intentions are only formed if farmers are aware of effective adaptation measures, accept self-responsibility, and evaluate adaptation costs positively (i.e. adaptation appraisal). While climate change-related risk and opportunity perception (i.e. climate change appraisal) support the formation of agricultural adaptation intention, our empirical data suggest that farmers’ adaptation appraisal is decisive for their intention to adapt.

We also find that most adaptation measures have been or are planned to be implemented due to a mix of climatic and non-climatic reasons such as availability and quality of natural resources, farm characteristics, farmers’ environmental attitude, and economic factors. Therefore, we suggest that engagement strategies to strengthen adaptation in agriculture should consider regional peculiarities, farm type-specific needs and challenges, farmers’ socio-cognitive processes, and adaptation costs and benefits. Promoting multi-purpose adaptation could be a promising option in order to increase adaptation intention even among farmers with only little climate change awareness, i.e. within the groups of integrative adaptors and cost-benefit calculators. Strengthening farmers’ environmental attitude could help to sensitize integrative adaptors not only for the environment, but also for climate change. Emphasizing potential on-farm and off-farm benefits and rewarding farmers’ responsibility for environment, nature and climate could be a starting point to evoke farmers’ adaptation intentions.

Planning security and financial support will play an important role, because economic factors were stressed for farmers’ adaptation decision-making, particularly within the group of cost-benefit calculators. Alternatively, this group could also respond to messages focusing on so-called ‘win-win’ technologies and practices, which offer economic rewards through an increase in efficiency. It is probably most difficult to convince the climate change fatalists of adaptation. However, communication and social interaction may reduce their unfounded risk perceptions and encourage adaptation action.

Following the mixed methods approach, in a next step, a standardized quantitative survey could be conducted in order to verify the farmer groups we have identified and to analyze their distribution in the total population of Austrian farmers. Similar to the approaches taken by Barnes and Toma 2012, Nguyen et al. 2016, and Rogers et al. 2012, survey items and response categories could be derived from the semi-structured interviews presented in this study to increase their comprehensibility and usefulness. Therefore, a questionnaire should be designed to cover cognitive elements as well as socio-environmental context factors as revealed by our results, e.g. beliefs in climate change, perceptions of climate change as risk or opportunity, knowledge and assessment of adaptation measures and self-responsibility. Results from a standardized follow-up survey could be the basis for assessing the relative relevance of the newly identified patterns of adaptation formation in agriculture and eventually enhance our results by identifying further groups of farmers on climate change perceptions and adaptation intentions (see e.g. Arbuckle et al. 2014; Barnes and Toma 2012; Eggers et al. 2015; Hyland et al. 2016). Finally, such statistically based groups of farmer could be used by scientists, e.g. to inform quantitative integrated assessments and behavioral farm models, and by agricultural knowledge brokers to tailor educational and outreach activities to farmers’ concerns and needs.

## Supplementary information

Supplementary material.
