# Supplementary material for: Exploring Farmers’ Climate Change Perceptions and Adaptation Intentions: Empirical Evidence from Austria
Source: Environ Manage. 2019 Apr 16;63(6):804–21. doi: 10.1007/s00267-019-01158-7 (PMC6525669; doi:10.1007/s00267-019-01158-7)
Supplement: Supplementary file 1 — Supplementary material. [file 267_2019_1158_MOESM1_ESM.docx]

**Exploring Farmers’ Climate Change Perceptions and Adaptation Intentions: Empirical Evidence from Austria**

# Supplementary material

**SM 1. Interview guide (translated from German)**

| **Introduction** |  | |  |
| --- | --- | --- | --- |
| - Acknowledgement for taking the time to contribute to the research project.  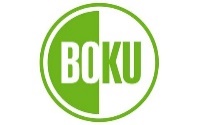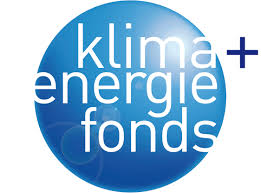  - Ask for permission to record the interview and use data for research project. - Summarize data privacy act and ask for signing consent form. - Interviewer signs and hands over protection of confidence form. - Explain expected duration of the interview: one hour. - Summarize aim of the research project. - Emphasize interest in the interview partners’ personal experience, perceptions & attitudes. - Provide overview on the interview guide. - Questions? | | | 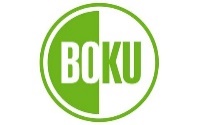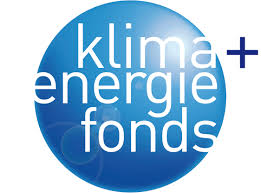 |
| **Guiding question** | | **Sub-question** | **Check/Memo/Examples** |
| **1.** Our project deals with climate change and its impacts on agriculture.  To start with, would you please share your view on climate change? | | What do you think is the humans’ role in climate change? | Belief in anthropogenic climate change |
| **2. Perceived changes in climate conditions**  Which changes in climate conditions do you already perceive in the region of Mostviertel/South-East Styria? | | *Perceived change 1:*  *Perceived change 2:*  *Perceived change 3:*  Which other changes in climate conditions do you perceive? | Temperature  Precipitation  Wind  Extreme weather events |
| **3. Perceived impacts on the farm**  You have mentioned *(Perceived change 1).* How does it affect your farm?  With respect to (*Perceived change 2, 3 etc.*), what impacts does it have on your farm? | | *Perceived impact 1/2/3/etc. related to change 1:*  *Perceived impact 1/2/3/etc. related to change 2:*  *Perceived impact 1/2/3/etc. related to change 3:*  Which other impacts on your farm do you perceive?  You mentioned *(Perceived impact 1/2/3).* Would you please share your thoughts on *(Perceived impact 1/2/3)?* | Production and income-related  Related to resources and ecology  Workflow  Administration |
| **4. Implemented adaptation measures**  How do you deal with (*Perceived impact 1/2/3/etc.)?*  Which other measures have you taken to deal with changes in climate conditions?  Which measures have you already taken in response to (*Perceived impact 1/2/3/etc.)?*  What are you doing about (*Perceived impact 1/2/3/etc.)?* | | *Implemented adaptation measure 1:*  *Implemented adaptation measure 2:*  *Implemented adaptation measure 3:*  **Evaluation of already implemented adaptation measures**  To what extent does *(Implemented adaptation measure 1/2/3/etc*.) meet your expectations?  To what extent are you satisfied with *(Implemented adaptation measure 1/2/3/etc*.)?  How well does *(Implemented adaptation measure 1/2/3/etc*.) work? | Adaptation measures, e.g., related to  Management (e.g., agronomic, financial, technical progress)  Investment  Cultivation system  Information and consultation  Effectiveness and efficiency of adaptation measures |
| **5. *If only risks or opportunities have been mentioned***  In the context of changing climatic conditions, positive (negative) impacts on farms are discussed as well.  Which positive (negative) impacts have you already perceived on your farm?  How do you deal with *(Positive/negative impact 1/2/3)?* | | *Perceived positive/negative impact 1:*  *Perceived positive/negative impact 2:*  *Perceived positive/negative impact 3:*  What are you doing in order to reduce this risk/take advantage of this opportunity? | Opportunities and risks |
| **6. What is the role of changes in climatic conditions for decisions on your farm?** | |  | Drivers and barriers  Adaptation intention  Avoidance |
| **7. Expected changes in climate conditions**  *We have already talked about current conditions. Now, I would like you to think about the future.*  Which changes in climate conditions are you expecting for the region of Mostviertel/South-East Styria in the future? | | *Expected change 1:*  *Expected change 2:*  *Expected change 3:* | Trend  Extreme weather event |
| **8.** **Expected impacts on the farm**  What do you think, how will *(Expected change 1/2/3)* affect your farm? | | *Expected impact 1/2/3/etc. related to expected change 1:*  *Expected impact 1/2/3/etc. related to expected change 2:*  *Expected impact 1/2/3/etc. related to expected change 3:*  Which other impacts on your farm do you expect from future changes in climate conditions?  You mentioned *(Expected impact1/2/3).* Would you please share your thoughts on *(Expected impact 1/2/3)?*  With respect to future changes in climate conditions, to what extent would you expect risks/opportunities for your farm? | Specific risks and opportunities related to future changes in climate conditions, e.g.,  Production and income-related (e.g., yield loss)  Related to resources and ecology (e.g., soil erosion)  Work flow (e.g., more/less work)  Administration |
| **9. Adaptation appraisal**  Which options do you consider as useful to successfully adapt your farm to changes in climate conditions? | | ***If few or no adaptation options are considered***  Why do you think are the options to adapt your farm to *(Expected changes 1/2/3)* limited?  Which other options do you perceive to deal with *(Expected impacts 1/2/3)?* | Potential adaptation measures  Perceived self-efficacy  Potential adaptation stimuli  Potential barriers to adaptation |
| **10. Barriers and stimuli**  What conditions should be met to successfully adapt your farm to changes in climate conditions?  Which barriers do you experience to successfully adapt your farm to changes in climate conditions?  What kind of support would you need to successfully adapt your farm to changes in climate conditions? | |  | Adaptation appraisal  Requirements  Potential adaptation stimuli  Potential barriers to adaptation (e.g., institutional, legal, administrative barriers, lack of infrastructure) |
| **11.** Would you like to **add something** we have not discussed yet? | |  |  |
| **Closing** | |  |  |
| - Many thanks for this interview. - Fill out standardized, one-page questionnaire. - Interested in final results? - Suggestions for interview partners?   Take notes (form). | | | |

**SM 2. Standardized one-page questionnaire (translated from German)**


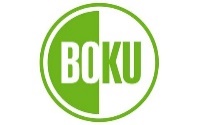

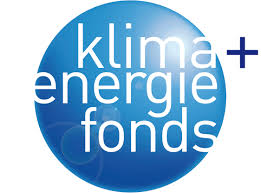


**Demographic and farm structural data:**

Project acronym: ___________________ Interviewer: ______________________

ZIP code: ___________________________ Date: ___________________

Interview code: ___________________ Duration of interview: ____________________

**Demographic data:**

Year of birth: _________

Gender: □ male □ female

**Farm structural data:**

Cropland: __________ ha Vegetables: __________ ha

Grassland: __________ ha Forest: __________ ha

Fruits: __________ ha Others: __________ ha

Vineyard: __________ ha

Own land: __________ ha Leased land: __________ ha

**Major crops in 2016:**

1. ____________________________ ______ ha
2. ____________________________ ______ ha
3. ____________________________ ______ ha
4. ____________________________ ______ ha

Livestock: □ yes □ no

Type of livestock Number in 2016:

1. ____________________________ _________
2. ____________________________ _________
3. ____________________________ _________
4. ____________________________ _________

Cultivation system:

□ Conventional □ Organic

□ Integrated □ In conversion, from ____________ to ____________

Full/part time farming:

□ Full time, since ____________ □ Part time, since ____________

**SM 3. Form for structured notes to be taken after the interview (translated from German)**


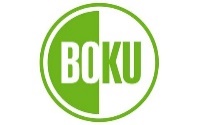

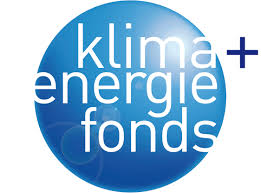


**Notes to semi-structured interviews with farmers**

Project acronym: ___________________ Interviewer: ______________________

ZIP code: ___________________________ Date: ___________________

Interview code: ___________________ Duration of interview: ____________________

- Interview atmosphere (e.g., mood, behavior of interview partner/s): ……………………………………………………………………………………………………………………………………………………………………………………
- Interaction between interviewer and interview partner(s)
  Interaction between interview partners: ………………………………………………………………………………………………………………………………………………………………………………………………………………………………………………………………………………
- Major topics (incl. relation to research questions):

………………………………………………………………………………………………………………………………………………………………………………………………………………………………………………………………………………

- Additional topics(incl. relation to research questions):

……………………………………………………………………………………………………………………………………………………………………………………

- Disturbances during the interview (e.g., kids, radio): ……………………………………………………………………………………………………………………………………………………………………………………
- Miscellaneous:

……………………………………………………………………………………………………………………………………………………………………………………

**SM 4. Sample description - summary of demographic data and farm structural data**

| # | Farming region | # of Intv | Codes | Gender | Age | Farm type | Major production activity | Cultivation system | Farm size (ha) | Full/part-time | Contact established via direct (D) or indirect (I) approach | |
| --- | --- | --- | --- | --- | --- | --- | --- | --- | --- | --- | --- | --- |
| 1 | Mostviertel | 1 | M1 | m | 48 | FC, PC | Crop and fruit production | C* | 53 | F | I | Gatekeeper |
| 2 | Mostviertel | 1 | M2 | w | 60 | LIVE | Dairy goats, goat cheese production | O | 30.7 | F | D | Online search |
| 3 | Mostviertel | 2 | M3,1/M3,2 | m/w | 52/52 | LIVE | Cattle | C | 30 | F | D | Interviewer's network |
| 4 | Mostviertel | 2 | M4,1/M4,2 | m/w | 49/47 | LIVE | Hog fattening, fruit and crop production | O | 47.7 | F | I | Gatekeeper |
| 5 | Mostviertel | 1 | M5 | m | 44 | FC, PC | Crop, fruit and cider production, direct marketing | O | 49.5 | F | D | Interviewer's network |
| 6 | Mostviertel | 1 | M6 | m | 36 | LIVE | Hog fattening, crop production | C | 65 | F | I | Snowball sampling |
| 7 | Mostviertel | 1 | M7 | m | 46 | LIVE | Dairy cows, direct marketing | C | 25 | P | D | Online search |
| 8 | Mostviertel | 1 | M8 | m | 44 | FC | Crop production | C | 263 | F | I | Gatekeeper |
| 9 | Mostviertel | 3 | M9,1/M9,2/M9,3 | m/w/m | 75/40/18 | LIVE | Hog fattening, crop production | C | 27 | P | D | Interviewer's network |
| 10 | Mostviertel | 3 | M10,1/M10,2/M10,3 | m/w/m | 50/49/26 | LIVE | Dairy cows, cheese production, direct marketing | O | 58 | F | D | Interviewer's network |
| 11 | South-East Styria | 1 | S1 | m | 49 | PC | Wine production, direct marketing incl. tavern | C/I | 41/36 | F | I | Gatekeeper |
| 12 | South-East Styria | 2 | S2,1/S2,2 | m/m | 61/27 | LIVE | Suckler cows and crop production | C | 30 | F | I | Gatekeeper |
| 13 | South-East Styria | 2 | S3,1/S3,2 | m/w | 66/54 | PC, FC | Fruit and crop production | I | 26.6 | F | I | Gatekeeper |
| 14 | South-East Styria | 1 | S4 | m | 57 | LIVE | Hog fattening, crop production | C* | 75.5 | F | I | Gatekeeper |
| 15 | South-East Styria | 1 | S5 | m | 53 | FC, PC | Crop and fruit production | I* | 29 | F | I | Gatekeeper |
| 16 | South-East Styria | 1 | S6 | m | 54 | LIVE | Hog fattening, crop production | C | 27.5 | F | I | Gatekeeper |
| 17 | South-East Styria | 1 | S7 | m | 42 | FC, PC | Crop and fruit production | O | 50.2 | P | I | Gatekeeper |
| 18 | South-East Styria | 1 | S8 | m | 33 | FC, PC | Field vegetables and fruit production | C | 68.5 | F | I | Gatekeeper |
| 19 | South-East Styria | 1 | S9 | m | 47 | LIVE | Cattle | C | 38.6 | P | D | Interviewer's network |
| 20 | South-East Styria | 2 | S10,1/S10,2 | m/w | 54/54 | LIVE | Hog fattening, bio-energy, composting | C | 53.4 | P | I | Gatekeeper |

*Intv* (interviewee)
Farm size in ha includes owned and leased land.
Farm type: *FC* field crop production, *PC* permanent crop production, *LIVE* livestock production
Cultivation system: *C* conventional, *C** conversion from conventional to organic, *C/I* partly conventional and integrated, *I* integrated, *I** conversion from integrated to organic,
*O* organic
